# Association between pseudoexfoliation and Alzheimer’s disease-related brain atrophy

**DOI:** 10.1371/journal.pone.0286727

**Published:** 2023-06-08

**Authors:** Won Cheol Jeong, Jin-Young Min, Tae Gu Kang, Heewon Bae

**Affiliations:** 1 Department of Neurology, Veterans Medical Research Institute, Veterans Health Service Medical Center, Seoul, Republic of Korea; 2 Veterans Medical Research Institute, Veterans Health Service Medical Center, Seoul, Republic of Korea; 3 Yonsei Bom Eye Clinic, Seoul, Kyeonggi-do, Republic of Korea; 4 Department of Clinical Research Design & Evaluation, Samsung Advanced Institute for Health Sciences and Technology, Sungkyunkwan University, Seoul, Republic of Korea; Bascom Palmer Eye Institute, UNITED STATES

## Abstract

**Background/aims:**

Pseudoexfoliation (PEX) syndrome is an age-related disorder characterized by the accumulation of extracellular material in the anterior eye segment. PEX pathogenesis is not fully understood, but amyloid which accumulates in the brain of patients with Alzheimer’s disease (AD) is a PEX component. PEX deposition shares features with amyloid aggregation in AD, and brain atrophy is a common AD feature, with β-amyloid accumulation among contributing factors. This study investigated whether PEX syndrome is associated with AD-related brain atrophy.

**Methods:**

We reviewed the medical records of patients diagnosed with PEX at the Veterans Health Service Medical Center between January 2015 and August 2021. This retrospective cohort study included 48 patients with PEX and 48 healthy age- and sex-matched controls. Patients with PEX were divided into two groups: with and without glaucoma. The main outcome measure was brain atrophy, using a visual rating scale, and AD incidence. Brain atrophy was measured using the Scheltens scale for medial temporal atrophy, the posterior cortical atrophy scale for parietal atrophy, and the Pasquier scale for global cortical atrophy.

**Results:**

The percentage of participants with medial temporal atrophy was 56.3% in the PEX group and 35.4% in the control group. The global cortical atrophy and parietal atrophy scores were significantly higher in the PEX group (P<0.05), whereas the PEX and PEX glaucoma groups showed no difference. Among the 96 participants, 16 and 5 participants in the PEX and control groups, respectively, were diagnosed with dementia. Patients with PEX glaucoma tended to have lower Mini-Mental State Examination scores, indicating impaired cognitive function, than those without glaucoma.

**Conclusion:**

PEX is associated with brain atrophy, reflecting the risk of developing AD. Patients with PEX glaucoma may present with advanced AD stages. Our results suggest that PEX may be a predictor of AD.

## Introduction

Pseudoexfoliation (PEX) syndrome is an age-related disorder characterized by the accumulation of extracellular material in the anterior segment of the eye [1]. PEX affects fibrillar deposition along the periphery of the lens and iris in the eye. The deposition of this material in the trabecular meshwork could be the cause of PEX glaucoma, resulting in aqueous outflow obstruction and increased intraocular pressure (IOP) [2]. PEX material has been found in various structures of visceral organs like the lung, liver, kidney, and gall bladder, as well as the cerebral meninges [3]. The pathogenesis of these deposits remains unclear, but previous studies have identified various extracellular matrix components, such as fibrillin-1 and fibulin-2, in PEX material [4, 5]. Abnormal elastic microfibril aggregation in PEX deposits shares some features with the accumulation of amyloid in Alzheimer’s disease (AD). Interestingly, amyloid has been identified in PEX material, and alpha-1 antichymotrypsin, a regulator of amyloid formation, has also been found [6]. Amyloid has also been found in the aqueous humor of patients with PEX [7].

AD is characterized by amyloid accumulation. This is caused by the production and deposition of β-amyloid (Aβ) peptides. There are two methods of Aβ accumulation: increased production due to genetic defects and failure of the clearance mechanism [8]. In addition, AD can be caused by neurofibrillary tangles, inflammation, and other changes in brain structure and function. Brain atrophy is a common finding in AD and can be caused by various factors including accumulation of Aβ [9]. Brain atrophy assessed using structural MRI is a valid marker of AD-related neurodegeneration confirmed by postmortem histology [10]. MRI-detected neurodegeneration can be used to support the clinical diagnosis of AD and to monitor disease progression [11]. Brain atrophy assessed using a comprehensive visual rating scale has predictive value in the diagnosis and progression of AD [12].

The pathogenesis of AD involves the spontaneous aggregation of Aβ monomers and deposition of Aβ oligomers, which are neurotoxic [8]. The deposition of amyloid has similar features to the elastotic process in PEX, in that such extracellular material leads to deterioration of the function of the eyes and brain. Two epidemiologic studies have investigated the association between AD and PEX, but the results are controversial. A 30-year follow-up study of 1,123 people found that PEX is not a predictor of AD [13]. In another study, 67 patients with PEX were selected as a control group matched by age, sex, and educational background to determine the presence or absence of AD. The results demonstrated that PEX is associated with high dementia prevalence [14].

Considering previous research, PEX and AD are associated, but there is no study on AD-related brain atrophy in patients with PEX. Thus, the current study aimed to evaluate whether PEX is associated with brain atrophy and AD.

## Materials and methods

### Study population

**Fig 1:**
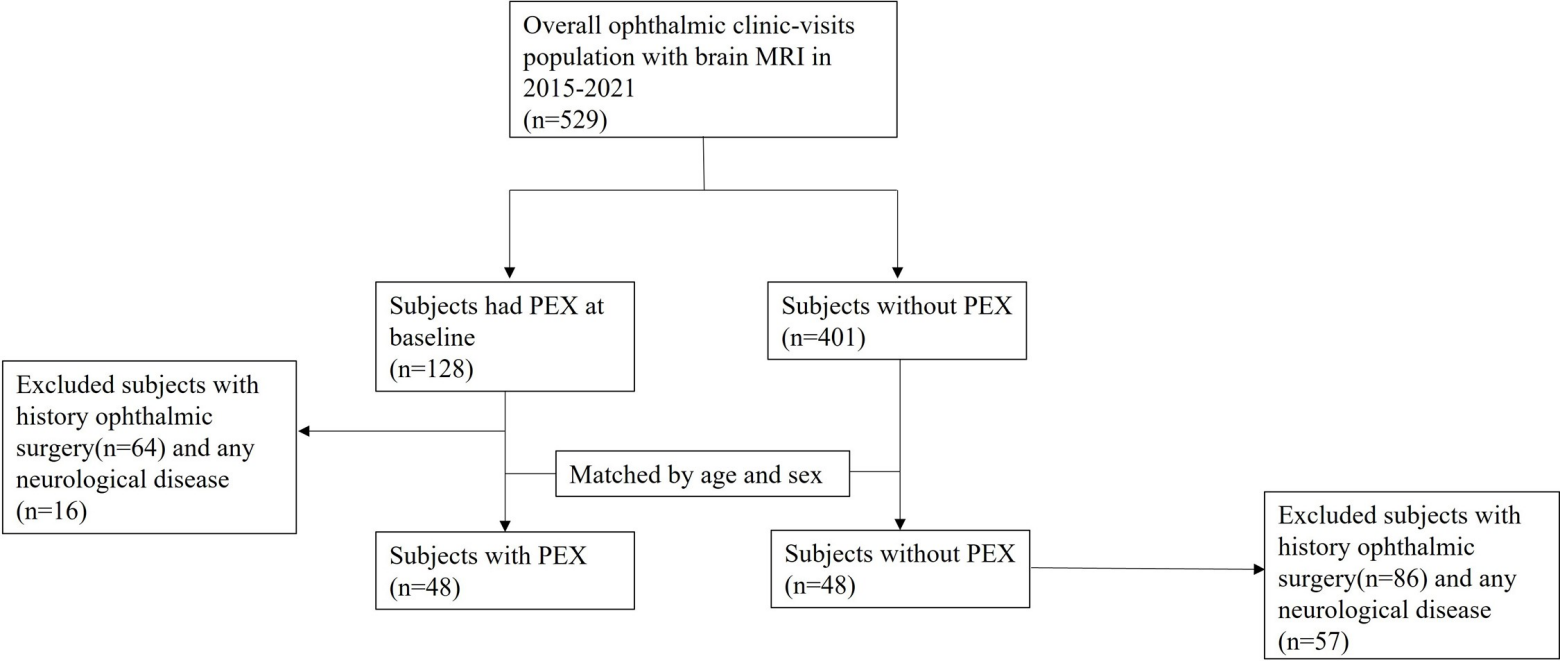
Flow chart showing the selection of the study population. The PEX group comprised 35 patients with PXS and 13 patients with PXG. *PEX*: *pseudoexfoliation; PXG*: *PEX glaucoma; PXS*: *PEX syndrome*.

This study enrolled participants who underwent ophthalmic examination and brain MRI between January 2015 and August 2021 at the Ophthalmologic Department of Veterans Health Service (VHS) medical center (n = 529). In total, 128 participants were diagnosed with PEX. Participants with a history of ophthalmologic surgery or disease that could affect PEX observation, and those with neurological disorders that could be a problem in the evaluation of AD, were excluded. After excluding participants with a history of ophthalmic surgery, ocular trauma, optic nerve atrophy, uveitis, diabetic retinopathy, age-related macular degeneration, or neurological disorders, such as stroke, dementia, or Parkinson’s disease, 48 participants were included in the study group. The control group consisted of 48 age- and sex-matched subjects who visited the VHS medical center for ophthalmic examinations. They were recruited as healthy persons among those without neurological diseases or ophthalmic abnormalities. The selection of the participants is depicted in Fig 1. All subjects underwent brain MRI within 1 year after detailed ophthalmic examination and were assessed for brain atrophy. We investigated patients with newly developed AD through follow-up after the occurrence of PEX.

This study was approved by the Institutional Review Board of VHS Medical Center. The Institutional Review Board of VHS Medical Center determined that informed consent was not necessary because previous medical records were reviewed. Patient information was anonymized and de-identified prior to the analysis.

### Pseudoexfoliation diagnosis

All subjects underwent a complete ophthalmic examination, including visual acuity testing using a Snellen chart, slit-lamp examination after pupil dilation, visual field examination, noncontact tonometry, central corneal thickness (CCT) measurement, optical coherence tomography, and retinal nerve fiber layer (RNFL) thickness measurement. Pupil dilatation was performed to evaluate the PEX material. PEX was defined as the presence of fibrillar deposits in the anterior segment of the eye. We considered subjects with PEX, regardless of whether the PEX deposits were unilateral or bilateral. PEX glaucoma (PXG) is severer than PEX syndrome (PXS), as the accumulation of PEX material can cause damage to the trabecular meshwork and reduce aqueous outflow resulting in PXG. Therefore, we classified the PEX group according to glaucoma diagnosed by an ophthalmologist as visual field defects and RNFL damage.

### Brain imaging

A 3-Tesla brain MRI (Skyra, Siemens, Germany) examination was performed within 1 year after the initial diagnosis of PEX. Two neurologists, who were unaware of the participants’ clinical statuses, evaluated the visual ratings of brain atrophy. The visual rating of medial temporal atrophy (MTA) was performed on oblique coronal T2-weighted images according to the Scheltens scale using the average score of the left and right sides (range 0–3). Parietal atrophy (PA) was rated using the posterior cortical atrophy scale (range 0–3). Global cortical atrophy (GCA) was evaluated on axial and coronal fluid-attenuated inversion recovery (FLAIR) images (range 0–3). The details regarding the scales mentioned can be found in Table 1.

**Table 1:** Visual rating scales of brain atrophy.

MTA (Scheltens et al.)
0 = normal
1 = widened choroid fissure
2 = increased width of the choroid fissure and temporal horn, opening of other sulci
3 = pronounced volume loss of the hippocampus
4 = end-stage atrophy
GCA (Pasquier et al.)
0 = no atrophy
1 = mild atrophy, opening of sulci
2 = moderate atrophy, volume loss of gyri
3 = severe atrophy, knife blade
PA (Koedam et al.)
0 = no atrophy
1 = mild atrophy, opening of sulci
2 = moderate atrophy, volume loss of gyri
3 = severe atrophy, knife blade

GCA: global cortical atrophy; MTA: medial temporal atrophy; PA: parietal atrophy.

### Dementia assessment

We reviewed medical records to identify subjects diagnosed with Alzheimer’s dementia. Participants were diagnosed with probable AD using the criteria of the National Institute for Neurological and Communicative Diseases Alzheimer’s Disease and Related Disorders Associations (NINCDS-ADRDA) [15]. All patients met the core clinical criteria of the National Institute on Aging-Alzheimer’s Association guidelines for AD. The core clinical criteria are as follows: 1. reduced ability to function at work or in usual activities; 2. decline in previous levels of functioning and performing; 3. absence of delirium or major psychiatric disorder; 4. assessment of cognitive impairment assessed through a combination of history taking from the patient and a knowledgeable informant and neuropsychological examination; and 5. cognitive and behavioral impairment involvement in two or more domains.

### Statistical analysis

Data are presented as means (standard deviations) for continuous variables and as numbers (percentages) for categorical variables. Comparisons of baseline characteristics between the two groups were performed using the independent t-test for continuous variables and the chi-square test for categorical variables. We compared the visual ratings of brain images according to the groups using the chi-square and Fisher exact tests. A P-value less than 0.05 (two-tailed) was considered statistically significant. All statistical analyses were performed using R3.5.1 (R Foundation, Vienna, Austria).

## Results

### Participants

The baseline characteristics of the patients are summarized in Table 2. This study included 96 participants, 48 in the PEX group and 48 in the control group. The mean age of the participants was 77.8 years, and the sex distribution was 15 women and 33 men in both groups. There were no significant differences in the comorbidities between the groups. The mean baseline IOP was 14.3±2.9 mmHg in the PEX group and significantly lower in the control group with 12.7±2.2 mmHg. Glaucoma was diagnosed in 13 patients with PEX.

**Table 2:** Clinical characteristics of participants included in the study.

	PEX (n = 48)	PXS (n = 35)	PXG (n = 13)	Control (n = 48)	P-value
Age (years)	77.8 (6.4)	77.9 (6.4)	77.5 (6.8)	77.8 (6.4)	>0.99
Sex					>0.99
Male	33 (68.8)	23 (65.7)	10 (76.9)	33 (68.8)	
Female	15 (32.3)	12 (34.3)	3 (23.1)	15 (32.3)	
Comorbidity					
Diabetes mellitus	16 (33.3)	10 (28.6)	6 (46.2)	14 (29.3)	0.83
Systemic hypertension	25 (52.1)	17 (48.6)	8 (61.5)	34 (70.1)	0.14
Social history					
Smoking					0.36
Ex-smoker	15 (31.3)	11 (31.4)	4 (30.8)	15 (31.3)	
Current smoker	2 (4.2)	0	2 (15.4)	0	
Drinking	8 (16.7)	5 (14.3)	3 (23.1)	17 (35.4)	0.1
Ophthalmologic findings					
IOP (mmHg)	14.3 (2.9)	14.4 (2.6)	14.3 (3.6)	12.7 (2.2)	0.01
CCT (μm)	551.8 (40.8)	551.3 (42.6)	553.0 (36.8)	532.9 (33.6)	0.01
RNFL thickness (mm)	77.9 (24.9)	83.1 (24.7)	72.2 (24.9)	88.6 (22.8)	0.17
Glaucoma	13 (27.1)				

CCT: central corneal thickness; IOP: intraocular pressure; PEX: pseudoexfoliation; PXG: PEX glaucoma; PXS: PEX syndrome; RNFL: retinal nerve fiber layer.

Thus, the PXS and PXG groups included 35 and 13 patients, respectively, and there was no difference in age between the two groups. The incidence rates for diabetes mellitus and systemic hypertension were higher in the PXG group than in the PXS group. Ophthalmologic findings showed no significant difference in IOP, but RNFL thickness was lower in the PXG group than in the PXS group.

P-values were calculated using the independent t-test or chi-square test for the comparison between PEX and control groups.

Data are presented as mean (standard deviation) or n (%).

### Comparison of brain atrophy in participants with or without PEX

**Fig 2:**
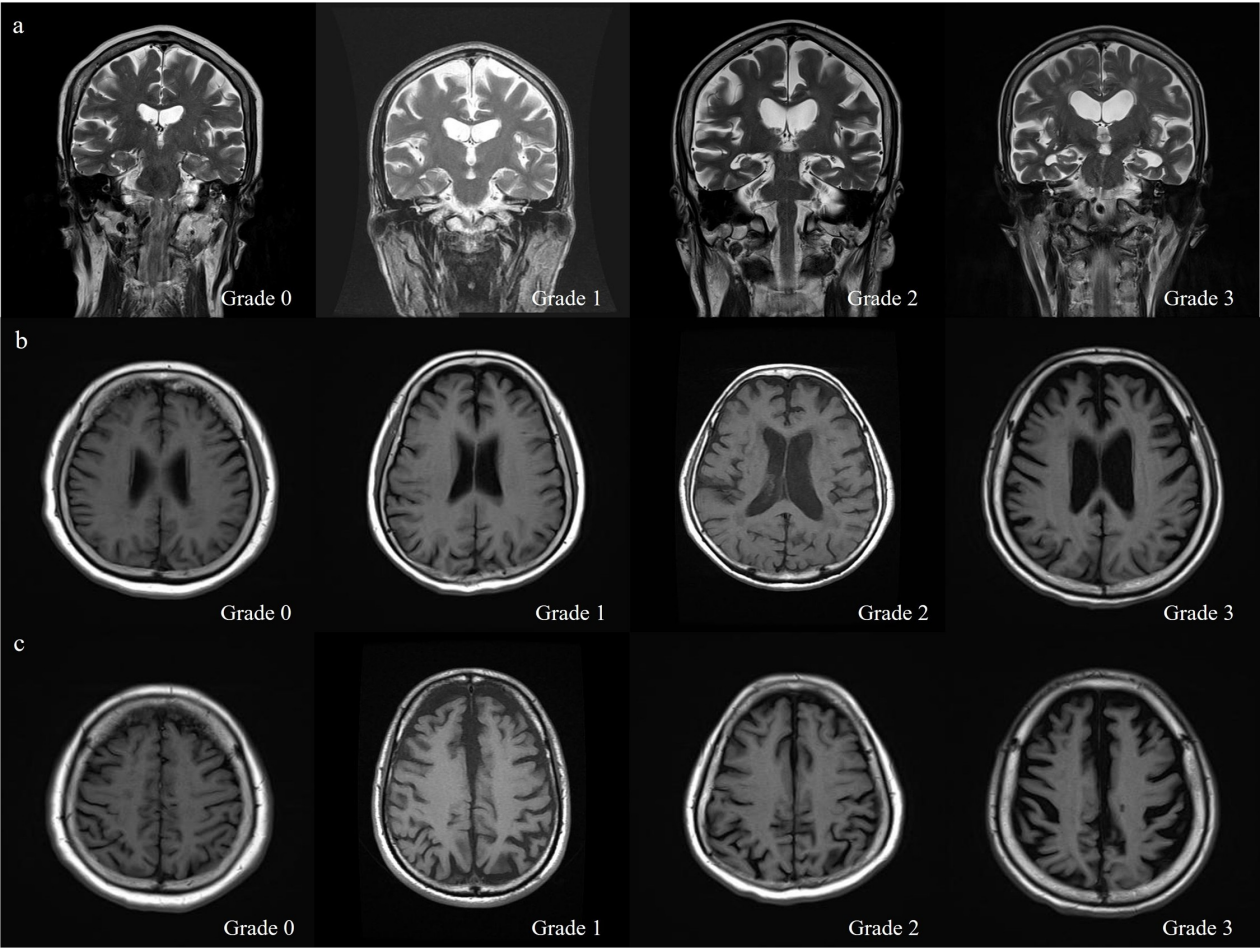
Brain atrophy in structural MRI using visual rating scales. (A) Medial temporal atrophy according to Scheltens scale with oblique coronal T2-weighted images. (B) Global cortical atrophy graded on axial FLAIR images. (C) Parietal atrophy scored using the posterior cortical atrophy scale with axial FLAIR images. *FLAIR*: *fluid-attenuated inversion recovery*.

The difference in MTA scores was not statistically significant, but the proportion of participants with MTA was 56.3% in the PEX group and 35.4% in the control group (Fig 2A). The GCA and PA scores were significantly higher in patients with PEX than in control participants (P<0.05; Fig 2B and 2C). There was also a statistically significant difference in GCA and PA scores in the PXS group and PXG group compared to the control group (both P<0.05). There was no difference in brain atrophy between the PXS and PXG groups (Table 3).

**Table 3:** Brain atrophy in the PEX and control groups.

	PEX (n = 48)	PXS (n = 35)	PXG (n = 13)	Control (n = 48)	P^a^	P^b^	P^c^
MTA					0.14	0.19	0.31
Grade 0	21	16	5	31			
Grade 1	19	15	4	12			
Grade 2	7	4	3	5			
Grade 3	1	0	1	0			
GCA					<0.05	<0.05	0.49
Grade 0	25	19	6	41			
Grade 1	18	13	5	6			
Grade 2	4	3	1	1			
Grade 3	1	0	1	0			
PA					<0.05	<0.05	0.41
Grade 0	12	9	3	23			
Grade 1	19	13	6	19			
Grade 2	16	13	3	6			
Grade 3	1	0	1	0			

P^a^ PEX compared with the control group

P^b^ PXG compared with the control group

P^c^PXS compared with PXG

GCA: global cortical atrophy; MTA: medial temporal atrophy; PA: parietal atrophy; PEX: pseudoexfoliation; PXG: PEX glaucoma; PXS: PEX syndrome.

### Rate of dementia in patients with PEX and control subjects

**Fig 3:**
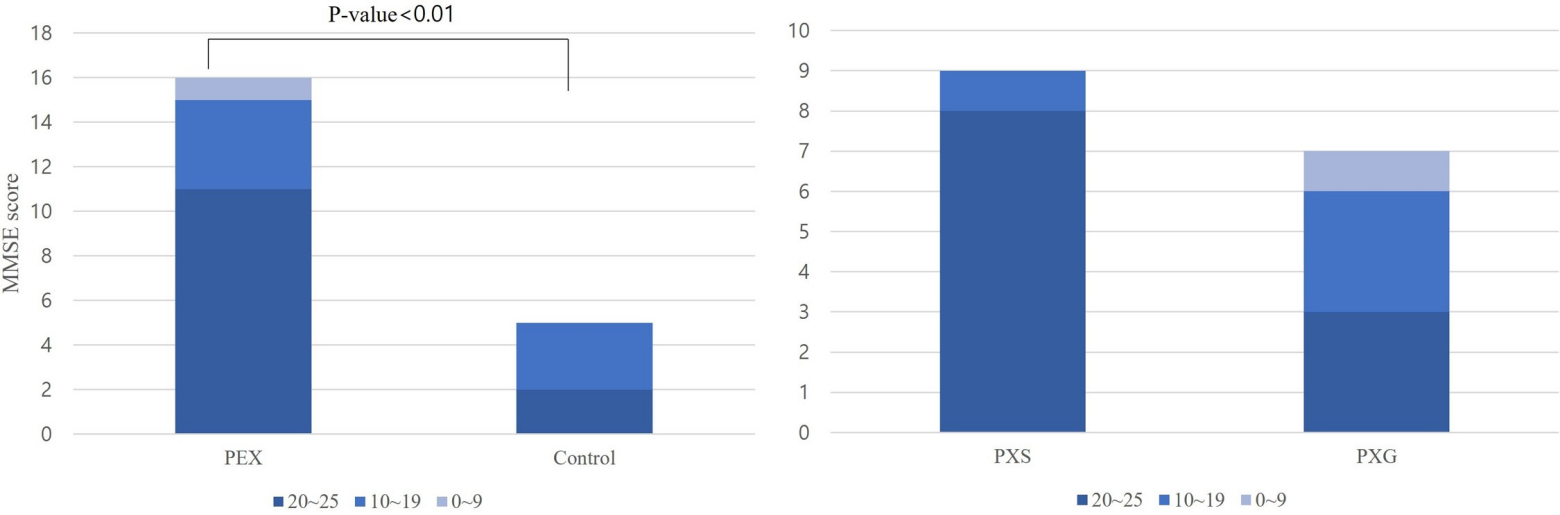
MMSE score distribution in patients with AD. (A) The PEX group had mainly mild MMSE scores in patients with AD. (B) The PXG group showed higher MMSE score severity than the PXS group. *AD*: *Alzheimer’s disease; MMSE*: *Mini-Mental State Examination; PEX*: *pseudoexfoliation; PXG*: *PEX glaucoma; PXS*: *PEX syndrome*.

Among the 96 participants, 16 and 5 patients in the PEX and control groups, respectively, were diagnosed with dementia. There was a statistically significant difference in the rate of AD (P<0.01). In total, 16 patients in the PEX group and 5 patients in the control group were newly diagnosed with AD according to the National Institute on Aging-Alzheimer’s Association guidelines. Patients were classified into three groups based on Mini-Mental State Examination (MMSE) scores (20–25, mild; 10–19, moderate; and 0–9, severe) according to severity. In the PEX group, 8 of 9 patients with AD showed mild scores, whereas in the PXG group, only 3 of 7 patients with AD showed mild scores, indicating that the severity of AD was greater in the PXG group than in the PXS group (Fig 3).

## Discussion

This study investigated the association between PEX and brain atrophy, a risk factor for AD. We found that the PEX group had significantly increased GCA and PA scores, and a higher proportion of MTA was related to AD. The PEX group also had a higher incidence of AD than did the control group. As a result of classifying severity according to glaucoma severity, the PXG group with high severity showed a lower MMSE score than the PXS group. These results suggest that PEX is associated with AD-related brain atrophy and can be an indicator of AD. Therefore, PXG may be indicative of advanced AD.

PEX is an age-related disorder in which extracellular material accumulates in the anterior segment of the eye and in organs other than the eye [16]. Although the mechanism of PEX material formation is not fully understood, histological analysis of 19 eyes in patients with PEX revealed amyloid deposits in the iris arterioles, lens capsule, and cornea [17]. It was also found that PEX material contains alpha-1 antichymotrypsin and alpha-1-antitrypsin, which are serine proteinase inhibitors that act as inflammatory substances and regulators of amyloid formation [6]. The deposition of amyloid in PEX has features similar to those in AD, which involves the accumulation of extracellular Aβ plaques in the brain. These observations have led to the suggestion of a possible relationship between PEX and AD.

Two previous studies investigated the epidemiological relationship between PEX and AD, with conflicting results. Ekström and Kilander reported the risk factors for AD in a cohort of 1,123 people over 30 years of follow-up and found using a Cox model that PEX did not increase the risk of AD. Although this was a large study involving 15,700 person-years, it had limitations in that it included medical records of patients who were undiagnosed [13]. In another study, Cumurcu et al. compared 67 PEX patients with 67 age-, sex-, and educational-background-matched control subjects. The results revealed that the frequency of AD was 67.2% in the PEX group and 26.9% in the control group, and this difference was significantly different [14]. The method was similar to that of our study, but unlike our study, which diagnosed AD through follow-up observation, it was a cross-sectional study. There was a difference in that the two previously mentioned studies diagnosed AD based on clinical symptoms, and our study evaluated AD based on brain MRI of the participants. Although the above studies do not provide a mechanism for the association between PEX and AD with consistent results, they suggest commonalities in the material deposit pathway. Amyloid and related substances were detected in PEX material, similar to their deposition in patients with AD [18]. A nerve growth factor that contributes to PEX formation and amyloid precursor protein expression has also been found in patients with PEX and those with AD [19, 20]. PEX material can leak into the aqueous humor via blood-derived proteins, breaking down the blood-aqueous barrier [5]. Amyloid was identified in the aqueous humor of patients with PEX [7]. Additionally, copper and zinc levels were elevated in the aqueous humor of patients with PEX and in the plaques of patients with AD [21, 22]. The production and absorption of aqueous humor and cerebrospinal fluid share some features [23]. The detection of amyloid in the aqueous humor of patients with PEX predicts an association between PEX and AD. These findings suggest that PEX may be associated with brain atrophy, a common feature of AD.

Brain atrophy can be a predictive factor for AD pathology [24]. Early detection of MTA predicts the progression from mild cognitive impairment to AD, and PA has also been shown in patients with preclinical AD [25, 26]. Brain atrophy was measured effectively using a visual rating scale. These results suggest that brain atrophy in the PEX group was associated with AD. The MMSE scores were lower in the PXG group than in the PXS group, indicating a higher severity of AD. This means that PEX accumulates and causes damage to the trabecular meshwork, and cognitive decline due to amyloid accumulation is more serious.

This study has some limitations. First, the population of patients who visited the Veterans Hospital may have introduced selection bias for participants who underwent ophthalmic examination and brain MRI. Second, the study included a relatively small number of patients. Therefore, future studies should be conducted with a more diverse and larger study population. Third, owing to the retrospective nature of the study, we could not measure the time-varying incidence of AD and could not check the MMSE score in all patients. Fourth, we only used a visual rating scale and did not measure the quantitative volume of the cortex. However, visual scaling of brain atrophy has shown reliable results in previous studies. Therefore, further studies should focus on establishing a standardized risk assessment for PEX-related dementia or the preclinical phase of dementia by using more research data.

## Conclusion

This study found a significantly higher incidence of brain atrophy and AD in patients with PEX compared to control participants. We suggest that PEX material in the anterior segments reflects amyloid accumulation in the brain. It can be considered that PXG, which is a severe form of PEX, is associated with advanced AD. Further investigations are required to determine the relationship between PEX and AD with a view to establishing PEX as a predictor of AD.

## Supporting information

S1 Data (XLSX)
